# Chronic Myeloid Leukemia with an e6a2* BCR-ABL1* Fusion Transcript: Cooperating Mutations at Blast Crisis and Molecular Monitoring

**DOI:** 10.1155/2017/9071702

**Published:** 2017-10-16

**Authors:** Mireille Crampe, Karl Haslam, Emma Groarke, Eileen Kelleher, Derville O'Shea, Eibhlin Conneally, Stephen E. Langabeer

**Affiliations:** ^1^Cancer Molecular Diagnostics, St. James's Hospital, Dublin 8, Ireland; ^2^Department of Haematology, St. James's Hospital, Dublin 8, Ireland; ^3^Department of Haematology, Bon Secours Hospital, Cork, Ireland; ^4^Department of Haematology, Cork University Hospital, Cork, Ireland

## Abstract

A minority of chronic myeloid leukemia patients (CML) express a variety of atypical* BCR-ABL1* fusion variants and, of these, the e6a2* BCR-ABL1* fusion is generally associated with an aggressive disease course. Progression of CML to blast crisis is associated with acquisition of additional somatic mutations yet these events have not been elucidated in patients with the e6a2* BCR-ABL1* genotype. Moreover, molecular monitoring is only sporadically performed in CML patients with atypical* BCR-ABL1* fusion transcripts due to lack of consensus approaches or standardization. A case of CML is described in which comprehensive molecular analysis, including targeted next-generation sequencing, revealed a single* ASXL1* mutation cooperating with an e6a2* BCR-ABL1* fusion transcript at blast crisis. A quantitative molecular monitoring approach was devised and adopted that reflected the disease response from initial treatment through allogeneic stem cell transplantation which resulted in undetectable e6a2* BCR-ABL1* transcripts. This case emphasizes the requirement for molecular monitoring in CML patients with atypical* BCR-ABL1 *fusion transcripts and emphasizes that comprehensive sequencing has the potential to identify targets for novel therapies in CML patients with advanced disease.

## 1. Introduction

The molecular genetic hallmark of chronic myeloid leukemia (CML) is the* BCR-ABL1* oncogene resulting from the t(9;22) translocation which fuses* ABL1* on chromosome 9q34 to* BCR* on chromosome 22q11. The vast majority of CML patients possess either e13a2 or e14a2* BCR-ABL1* fusion transcripts; however, several alternative transcripts have been reported that are largely the result of either* BCR* or* ABL1* alternative exon splicing. These uncommon, variant transcripts can result in phenotypic variability and affect response to tyrosine kinase inhibitor (TKI) therapy [1]. Since the initial molecular characterization of the e6a2* BCR-ABL1* transcript that fuses* BCR* exon 6 to* ABL1* exon a2 [2], less than twenty CML cases have been reported in which the e6a2 fusion is usually associated with a clinically aggressive disease frequently presenting in accelerated or blast crisis phases [3–6]. This transcript type has also been reported in t(9;22)-positive acute myeloid leukemia (AML) and transformed chronic myelomonocytic leukemia [7–9]. Although responses to imatinib have been reported [10, 11], several cases of* ABL1* kinase domain mutation-associated imatinib resistant, e6a2* BCR-ABL1* CML have been documented [12, 13] with limited information on the efficacy of front line second-generation TKIs in this genotype. As e6a2* BCR-ABL1* CML is associated with aggressive disease, allogeneic stem cell transplantation (ASCT) remains the only curative option and in such circumstances conventional AML therapy has been combined with a second-generation TKI as a bridge to ASCT [14–16].

Acquisition of further cytogenetic abnormalities and AML-associated, disease driving mutations are common findings in blast crisis of CML [17] but whether these additional events impact on prognosis is unclear [18]. Analogous to AML, identification of cooperating mutations may establish targets for novel therapeutic interventions in addition to TKI therapy [19]. Information on the incidence and pattern of these cooperating mutations in CML patients with the rare fusion transcript types is lacking.

Molecular monitoring of* BCR-ABL1* transcripts by reverse transcription-quantitative polymerase chain reaction (RT-qPCR) is an integral part of the modern management of CML patients with achievement of molecular milestones according to internationally standardized practice incorporated into current management guidelines [20, 21]. However, standardization of these techniques applies only to the common e13a2 and e14a2* BCR-ABL1* breakpoints with quantitative* BCR-ABL1* levels reported on an ad hoc basis in those patients with the rare, variant* BCR-ABL1* fusions.

A case of e6a2* BCR-ABL1* CML presenting in blast crisis is reported in which a targeted next-generation sequencing (NGS) approach was utilised to ascertain additional cooperating mutations. Furthermore, an RT-qPCR approach was developed to prospectively monitor the atypical* BCR-ABL1* transcripts patient throughout the disease course.

## 2. Case Report

**Figure 1:**
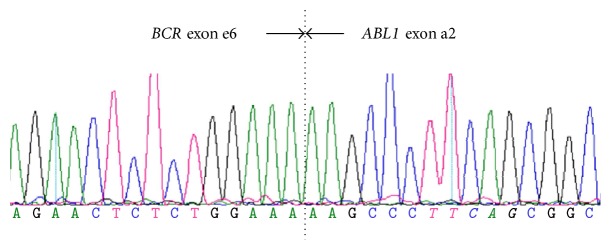
Sanger sequencing of* BCR-ABL1* breakpoint demonstrating fusion of* BCR* exon 6 to* ABL1* exon a2.

A 48-year-old female presented with a leucoerythroblastic blood film, anemia (hemoglobin 10.9 g/dL), thrombocytopenia (72 × 10^9^/L), normal white cell count, and no splenomegaly. An initial bone marrow (BM) aspirate was hypocellular with increased fibrosis suspected as the underlying cause. The* JAK2* V617F and* CALR* exon nine insertion or deletion mutations were not detected but cytogenetic analysis revealed a karyotype of 46,XX,t(9;22)(q42;q11) in 16 metaphases, trisomy 8 in a further two Ph+ metaphases, and tetrasomy 8 in one further Ph+ metaphase. RT-qPCR analysis with standardized primers and probes [22] did not detect* BCR-ABL1* transcripts but further analysis with a qualitative PCR approach [23] detected a single abnormal band which Sanger sequencing demonstrated to be a fusion of* BCR* exon 6 to* ABL1* exon a2 (Figure 1). No mutations were detected in the* ABL1* kinase domain. Immunophenotyping of the peripheral blood (PB) demonstrated 12% myeloblasts that were positive for CD7, CD13, CD33, CD34, CD117, and HLADR and negative for CD3, CD4, CD8, CD11c, CD14, CD15, CD19, and CD64. A repeat BM aspirate was hypercellular with 18% myeloblasts morphologically. The corresponding trephine biopsy was hypercellular and had prominent grade three fibrosis with CD34+ blasts accounting for approximately 40% of the total cellularity, consistent with a diagnosis of blast crisis CML with marrow fibrosis.

In order to quantitate e6a2* BCR-ABL1* transcripts, a* BCR* exon 6 forward primer [4] was used with reverse primer ENR561 and probe ENP541 [22]. A standard curve was established by tenfold dilutions of patient presentation PB cDNA (slope −3.538, *r*^2^ 0.996). Control* ABL1* transcripts were detected as previously described [22]. At diagnosis the patients' PB* BCR-ABL1*/*ABL1* transcript level was 78.6%.

An NGS approach was employed to detect additional mutations cooperating with the e6a2* BCR-ABL1* fusion in driving blast crisis in this patient. Amplicon libraries covering 30 commonly mutated genes implicated in myeloid malignancies either covering the entire coding region (*CALR*,* CEBPA*,* ETV6*,* EZH2*,* RUNX1*,* SH2B3*,* TET2*,* TP53*, and* ZRSR2*) or mutational hotspots (*ABL1*,* ASXL1*,* BRAF*,* CBL*,* CSF3R*,* DNMT3A*,* FLT3*,* GATA2*,* IDH1*,* IDH2*,* JAK2*,* KIT*,* KRAS*,* MPL*,* NPM1*,* NRAS*,* PTPN11*,* SETBP1*,* SF3B1*,* SRSF2*, and* U2AF1*) were generated using 20 ng BM genomic DNA and sequencing performed using Ion AmpliSeq™ methodology (Thermo Fisher Scientific, Paisley, UK). Calling of somatic mutations was achieved using an algorithm that excluded synonymous mutations, variants located within intronic or untranslated regions, and those present at a variant allele frequency (VAF) of <5%. A minimum target depth of coverage for variant calls was set at 500x as previously described [24]. A single* ASXL1*  p.E1102D mutation (c.3306G > T; reference sequence NM_015338.5) was detected with a variant allele frequency of 45.5%.

**Figure 2:**
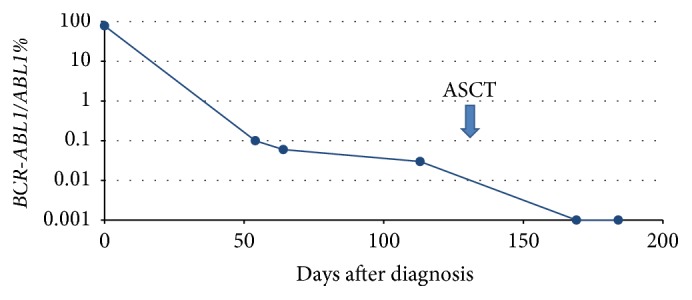
RT-qPCR of e6a2* BCR-ABL1* transcripts throughout clinical course. ASCT: allogeneic stem cell transplantation.

The patient received one cycle of daunorubicin and cytarabine (DA 3 + 10) with imatinib 400mg daily which was escalated to 600 mg daily achieving hematological and morphological remission and resulting in a PB* BCR-ABL1/ABL1* transcript level of 0.06%. She was subsequently treated with a second cycle of chemotherapy (DA 3 + 8) which was complicated by septicemia. Prior to ASCT the PB* BCR-ABL1/ABL* transcript level was 0.03% (Figure 2). Her imatinib was stopped prior to busulfan and cyclophosphamide conditioning for ASCT from a matched sibling donor that was complicated by mucositis and a coagulase-negative staphylococcus infection during her inpatient stay. After discharge she developed grade four skin graft versus host disease (GVHD) and was treated with high dose steroids subsequently developing CMV reactivation and steroid induced diabetes. Antiviral treatment with valganciclovir caused a pancytopenia requiring treatment with granulocyte colony-stimulating factor.* BCR-ABL1* transcripts were not detected by RT-qPCR at day 38 after ASCT with full donor chimerism achieved (100% at day 43 post-ASCT). The patient remains off TKI and generally well at last follow-up. The CMV reactivation has resolved and she continues on steroid taper for GVHD. Continued close RT-qPCR monitoring is planned.

## 3. Discussion

The e6a2* BCR-ABL1* variant is rare with less than twenty CML patients of this genotype reported. Clinically, CML cases that express the variant e6a2* BCR-ABL1* fusion transcript often present in advanced stages and display an aggressive disease course, confirmed by this case report: it must be noted that occasional good responses to TKI have also been documented. In the case described herein, imatinib combined with AML induction therapy resulted in a considerable reduction in the* BCR-ABL1* transcript level with the patient able to proceed with ASCT.

Little is known of the biological characteristics of this genetic subtype of CML and in those with other variant* BCR-ABL1* fusion genes. NGS targeted for prognostically and clinically relevant mutations recurrently observed in myeloid malignancies revealed a mutation of* ASXL1* in this patient.* ASXL1* encodes an epigenetic regulator involved in posttranslational chromatin modification with aberrant histone modification being one of the important mechanisms underlying altered epigenetic regulation in other myeloid malignancies such as the myelodysplastic syndromes (MDS) [25]. Mutations of* ASXL1* and other genes involved in histone modification and DNA methylation have been characterized in chronic phase CML patients but appear to be more frequent in accelerated or blast crisis phases [26–28]. Novel therapies currently under investigation, which specifically target aberrant DNA methylation and chromatin remodelling in MDS [29], are potentially applicable to other* ASXL1*-mutated malignancies such as blast crisis CML, suggesting that individual molecular characterization of advanced phase CML patients may be increasingly necessary. It must be acknowledged that mutations of* ASXL1* and other myeloid malignancy-associated genes have been detected at low levels by deep sequencing in both Ph-negative and Ph-positive clones of CML patients. These findings suggest that expression of* BCR-ABL1 *may be insufficient for the development of CML which has implications for treatment with both TKIs and other novel agents [18, 30]. Whether the* ASXL1* mutation detected in this case was present in a* BCR-ABL1*-independent clone cannot be excluded or confirmed as NGS was not performed when the patient had achieved remission after chemotherapy; however, the high* ASXL1* mutation VAF at presentation points to the* ASXL1* mutation and* BCR-ABL1* fusion coexisting in the same clone.

BM fibrosis, as evident in this case, is an infrequent but recurrent feature of CML. In the classical Philadelphia chromosome-negative myeloproliferative neoplasms, several lines of evidence link* ASXL1* mutations with BM fibrosis:* ASXL1* mutations are associated with a higher degree of BM fibrosis in primary myelofibrosis [31];* ASXL1* mutations are associated with an increased risk of myelofibrotic transformation in patients with essential thrombocythemia and polycythemia vera [32]; and* ASXL1* mutations are more frequently detected in overt as opposed to prefibrotic primary myelofibrosis [33]. An association therefore potentially exists between* ASXL1* mutations and BM fibrosis that requires further investigation in CML patients.

Despite the fact that there is no standardized approach to monitoring atypical* BCR-ABL1* transcript types, RT-qPCR has been adopted in some e6a2* BCR-ABL1* cases of CML correlating well with clinical course as in the case described herein. An emerging, alternative methodology to RT-qPCR is digital droplet PCR that allows for more accurate quantitation of target molecules without the use of standard curves. This approach has recently been applied to molecular monitoring of a CML patient with an e6a2* BCR-ABL1 *fusion, demonstrating an initial three-log reduction in transcripts with dasatinib monotherapy [34].

In conclusion, comprehensive molecular genetic characterization of the rare e6a2* BCR-ABL1* fusion and accompanying mutations facilitates prospective molecular monitoring and may also provide targets for potential therapeutic intervention in CML patients with this hostile genotype.
